# Selected Papers on Stone, Prostate, and Other Urinary Disorders

**Published:** 1900-06

**Authors:** 


**Selected Papers on Stone, Prostate, and other Urinary Disorders.** By REGINALD HARRISON, F.R.C.S. Pp. 190. London: J. & A. Churchill. 1899.

Several valuable papers by the author have appeared on the subject of urinary disorders since the last edition (1893) of his work on the *Surgical Disorders of the Urinary Organs*, and the present book is composed of these articles which have been brought up to date. The chapters are full of important suggestions, bearing the weight and authority of one so well known in this branch of surgery. The author's views on vasectomy for prostatic enlargement are well known, and he formulates the indications for this operation by suggesting its use in those cases of obstruction caused by the prostate where the enlargement is general and not limited to a pendulous lobe, and where the gland, from its feel, has not become largely fibrotic, but apparently retains a fair proportion of its secreting and muscular elements. The book is most instructive, and its chapters record a considerable advance in the ordinary treatment of affections of the kidneys and bladder.

